# Polypharmacy and potentially inappropriate medications (PIMs) in older adults referred to a memory clinic

**DOI:** 10.1192/bjo.2021.810

**Published:** 2021-06-18

**Authors:** Anietie Akpan, Bruno De Blaquiere, Issadevi Nellaya, Cornelia Termure, Sujoy Mukherjee

**Affiliations:** West London NHS Trust

## Abstract

**Aims:**

The older adult is more likely to be prescribed a lot of medications (polypharmacy) on account of multi-morbidity and consequently being under the care of several specialists. Adverse drug events and reactions account for significant morbidity and mortality in this population group. Common sequelae include confusional episodes, dementia syndromes, falls, and higher rates of acute hospital admissions.

Medications are not routinely reviewed in elderly care. We sought to estimate the prevalence of polypharmacy, and potentially inappropriate medications (e.g. anticholinergics or medications with central anticholinergic effects) in those referred to the Cognitive Impairment and Dementia Service (Elm Lodge), Older Persons Mental Health, West London NHS Trust.

**Method:**

All referrals between 01/10/2020 and 30/11/2020 were screened for medications prescribed. Polypharmacy was defined as prescription of 5 or more medications. Medications with anticholinergic properties were considered examples of Potentially Inappropriate Medications (PIMs). The Anticholinergic Effect on Cognition (AEC) Tool, ‘Medichec’, was used to identify and rate anticholinergic burden. Anticholinergic load was also compared using the Anticholinergic Burden Scale (ABS).

**Result:**

Total number of patients referred – 193

11 patients excluded due to unavailable/incomplete medication records.

Study number: 182

Polypharmacy:

- 79.67% (n = 145) were prescribed 5 or more medications.
- 44.51% (n = 81) prescribed 5–9 medications.
- 23.08% (n = 42) prescribed 10–14 medications.
- 8.79% (n = 16) prescribed 15–19 medications.
- 1.67% (n = 3) prescribed more than 20 medications.

Anticholinergics prescribed (AEC Tool):

- 37.36% (n = 68) prescribed an anticholinergic.
- 6.59% (n = 12) prescribed more than 1 anticholinergic.

Anticholinergics (ABS):

- 29.67% (n = 54) prescribed an anticholinergic.
- 7.699% (n = 14) prescribed more than 1 anticholinergic.

**Conclusion:**

Polypharmacy and potentially inappropriate prescribing (e.g. anticholinergics) remain widespread within the older adult population. Anticholinergic load was broadly similar with the Anticholinergic Effect on Cognition tool and the Anticholinergic Burden Scale. Increased anticholinergic burden further compounds risks of cognitive impairment, delirium and death.

Other categories of Potentially Inappropriate Medications, including those no longer needed, ought to be identified and reviewed. Over-the-counter medications also need to be screened for.

Elimination or reduction of anticholinergic burden may improve quality of life for patients, as well as cost burden on services.

Pharmacovigilance, collaborative working, and regular training are needed across services providing care for the older adult.

